# A Global Orientation Map in the Primary Visual Cortex (V1): Could a Self Organizing Model Reveal Its Hidden Bias?

**DOI:** 10.3389/fncir.2016.00109

**Published:** 2017-01-05

**Authors:** Ryan T. Philips, V. Srinivasa Chakravarthy

**Affiliations:** Computational Neuroscience Laboratory, Department of Biotechnology, Indian Institute of Technology MadrasChennai, India

**Keywords:** radial bias, retinotopy, meridional preference, orientation preference, self-organizing, LISSOM, V1, plasticity

## Abstract

A remarkable accomplishment of self organizing models is their ability to simulate the development of feature maps in the cortex. Additionally, these models have been trained to tease out the differential causes of multiple feature maps, mapped on to the same output space. Recently, a Laterally Interconnected Synergetically Self Organizing Map (LISSOM) model has been used to simulate the mapping of eccentricity and meridional angle onto orthogonal axes in the primary visual cortex (V1). This model is further probed to simulate the development of the radial bias in V1, using a training set that consists of both radial (rectangular bars of random size and orientation) as well as non-radial stimuli. The radial bias describes the preference of the visual system toward orientations that match the angular position (meridional angle) of that orientation with respect to the point of fixation. Recent fMRI results have shown that there exists a coarse scale orientation map in V1, which resembles the meridional angle map, thereby providing a plausible neural basis for the radial bias. The LISSOM model, trained for the development of the retinotopic map, on probing for orientation preference, exhibits a coarse scale orientation map, consistent with these experimental results, quantified using the circular cross correlation (*r*_*c*_). The *r*_*c*_ between the orientation map developed on probing with a thin annular ring containing sinusoidal gratings with a spatial frequency of 0.5 cycles per degree (cpd) and the corresponding meridional map for the same annular ring, has a value of 0.8894. The results also suggest that the radial bias goes beyond the current understanding of a node to node correlation between the two maps.

## Introduction

Hyper-columns in V1, mapping the entire range of orientations possible, have a flattened spatial extent of ~1 × 1 mm and ~2 × 2 mm, in monkeys and humans respectively (Blasdel and Salama, 1986; Adams et al., 2007). It would seem improbable that from Blood Oxygen Level Dependent—functional Magnetic Resonance Imaging (BOLD-fMRI) activity, which has a spatial resolution of ~3 × 3 × 3 mm corresponding to each voxel, one could successfully decipher the orientation of a grating pattern presented to a subject. However, landmark BOLD-fMRI results demonstrate that this is indeed possible (Kamitani and Tong, 2005; Sasaki et al., 2006). There are two prevalent hypotheses which attempt to explain these results: (a) Hyper-acuity: There exists a sampling bias in the population activity of hyper-columns that contribute to each fMRI voxel, which on multivariate pattern analysis would yield orientation discriminability (Kamitani and Tong, 2005; Haynes and Rees, 2006); (b) Coarse scale orientation maps: There exists an orientation map at a spatial scale equivalent to that of the retinotopic map. These maps have a radial bias, i.e., those orientations which match the retinotopic meridional angle are over-represented as compared to other orientations (Sasaki et al., 2006; Freeman et al., 2011). (The projection of retinal space onto the cortical space is called retinotopy. Meridional angle refers to the angular position of a stimulus with respect to the point of fixation, in radial co-ordinates.)

Recent studies, interpreting both psychophysics (Hong, 2015) as well as fMRI results, have confirmed the existence of a coarse orientation map which is radially biased (Sasaki et al., 2006; Freeman et al., 2011, 2013; Alink et al., 2013). The neural basis for the radial bias has been supported by electrophysiological recordings in the cat V1 (Leventhal and Schall, 1983; Schall et al., 1986). However, most studies at a population level, using techniques such as optical imaging, have not reported the presence of such a bias, either due to the limited spatial extent considered as speculated by Freeman et al. (2011), or due to the deliberate filtering out of global orientation biases (Shmuel and Grinvald, 1996). This being said, there is still debate regarding the necessity of these global orientation biases for orientation discriminability. Freeman et al. (2011) demonstrated that the orientation decoding accuracy reduces significantly when the orientations corresponding to the angular positions were removed prior to the classification, suggesting the necessity of the radial bias. However, Alink et al. (2013), have shown that fine-grained components of the fMRI activity also contribute to the orientation discriminability. A recent modeling effort by Carlson (2014) similarly suggests that any sort of bias is unnecessary for orientation decoding. Another debate in the field is regarding the successful decoding of anticlockwise vs. clockwise spiral gratings. These grating have equal local orientation edges at every radial spatial location. Mannion et al. (2009) speculate that this result negates the need for the radial bias and any coarse scale map in orientation decoding, since the fMRI responses in response to local features appear to be sufficient to account for the orientation decoding. However, subsequent experiments by Freeman et al. (2013), demonstrate the presence of a coarse scale bias even in response to these spirals. These studies primarily question the necessity of the radial bias in decoding orientations, not the existence of the radial bias itself. Secondly, the radial bias is conventionally quantified by the voxel to voxel correlation between the meridional and the orientation maps. The absence of such a correlation may be mistakenly construed to imply the absence of a radial bias.

The mechanisms involved in the development of the radial bias are not entirely understood. There is speculation that the radial bias might be a natural consequence of the retinal development as retinal ganglion cells are tiled in a radial fashion (Leventhal and Schall, 1983). The elongation of dendritic fields of Retinal Ganglion Cells toward the fovea could also contribute to the radial bias (Rodieck et al., 1985; Schall et al., 1986). When eye movements are directed toward a given position, radial orientations do not appear blurred, suggesting that eye movements may also play a role in the development of such a bias Sasaki et al. (2006). Recent results by Alink et al. (2013) hint at the possibility of lateral connections in V1 as well as top down effects playing a role in the development of global preference maps. Another series of experiments performed by Cichy et al. (2015) utilize MEG signals in order to probe orientation discriminability. The MEG signals obtained in the first 150 ms post stimulus presentation gave the highest discriminability across all stimuli considered. The radial bias has been implicated in explaining a number of experimental phenomena such as binocular disparity (Durand et al., 2002), context assimilation (Alexander et al., 2004), and perceptual distortions (Westheimer, 2003).

We propose that, given the appropriate training stimuli, there is a co-evolution of a large scale orientation and meridional map, resulting in a strong correlation between the two maps. The model described is the same as that used to develop the retinotopic map in Philips and Chakravarthy (2015) having 2 layers, one representative of the retina and the other representative of the primary visual cortex (V1). Each node in the output layer (V1) receives afferent inputs from the retinal layer, as well as recurrent lateral excitatory and inhibitory inputs from other nodes in V1. A detailed description of the architecture is provided in the Methods section.

We train the model now using rectangular bars having an aspect ratio of 0.025, which are near radial. These stimuli now also cause the nodes of the V1 layer to have a preference toward radial orientations. This is because each node in the V1 layer, trained exclusively on near radial edges (because the bars are centered), using the Hebbian learning rule, evolves a receptive field which prefers such edges. Now when the orientation preference of each node of V1 is probed, a coarse scale orientation map emerges.

The rationale for such a training regime is as follows: While it is true that external visual stimuli could have all possible orientations at all possible spatial locations, radial stimuli would result in larger activation of RGCs. This is due to the fact that the dendrites of the RGCs have their projective fields elongated toward the fovea (Rodieck et al., 1985; Schall et al., 1986). Thus, a radial or near radial edge would result in the co-activation of a larger number of inputs from the photo-receptors via the bipolar cells, which project to a particular RGC.

The exact radial bias in the retinal space is difficult to ascertain, since for different intensities of the actual visual stimulus, the retinal bias would vary. For example when the input stimuli are at the threshold of detection only radial stimuli would cause the RGCs to fire. We have performed a few additional simulations on varying the retinal radial bias, by introducing non-radial stimuli in the training regime as well. We demonstrate that for different ratios of radial to non-radial stimuli a reasonable cross correlation between the orientation and meridional maps still exists.

In order to validate the results of the model, the meridional and orientation preference maps are compared in a similar fashion as in Sasaki et al. (2006) and Freeman et al. (2011). It is observed that the model mimics the experimental results of the radial bias reasonably well. On increasing the spatial frequency of the gratings and the width of the annulus used for probing, the clean pixel to pixel correspondence between the meridional and orientation maps reduces (as reflected by the cross correlation scores); however the orientation map developed is still robust enough to achieve orientation discriminability. Thus, the insights gained from this model could possibly help reconcile contradictory experimental results which correlate the apparent presence or absence of a global orientation map and orientation discriminability.

## Methods

### LISSOM model

Self organizing mechanisms have been utilized extensively in explaining the development of cortical maps. The basic Self Organizing Map (SOM) model involves iteratively selecting winner nodes which maximally respond to a particular input stimuli and then updating the weights of nodes within a defined radius around the winning node (Kohonen, 1982, 1990). A variant of the SOM model, namely LISSOM (Sirosh and Miikkulainen, 1994; Miikkulainen‘ et al., 2005), wherein the self-organization is achieved by including lateral connections between nodes has been deemed more biologically realistic and has been invoked to explain a number of experimentally observed phenomena in the visual cortex (Bednar, 2012). A recent implementation of the LISSOM model has been used to develop a large scale retinotopic map which closely resembles the mapping of eccentricity and meridional angle (angular position) on to orthogonal axes in the V1 (Philips and Chakravarthy, 2015). This very model is utilized in the present study and built upon in order to investigate a plausible theory for the development of the radial bias seen in V1.

**Figure 1:**
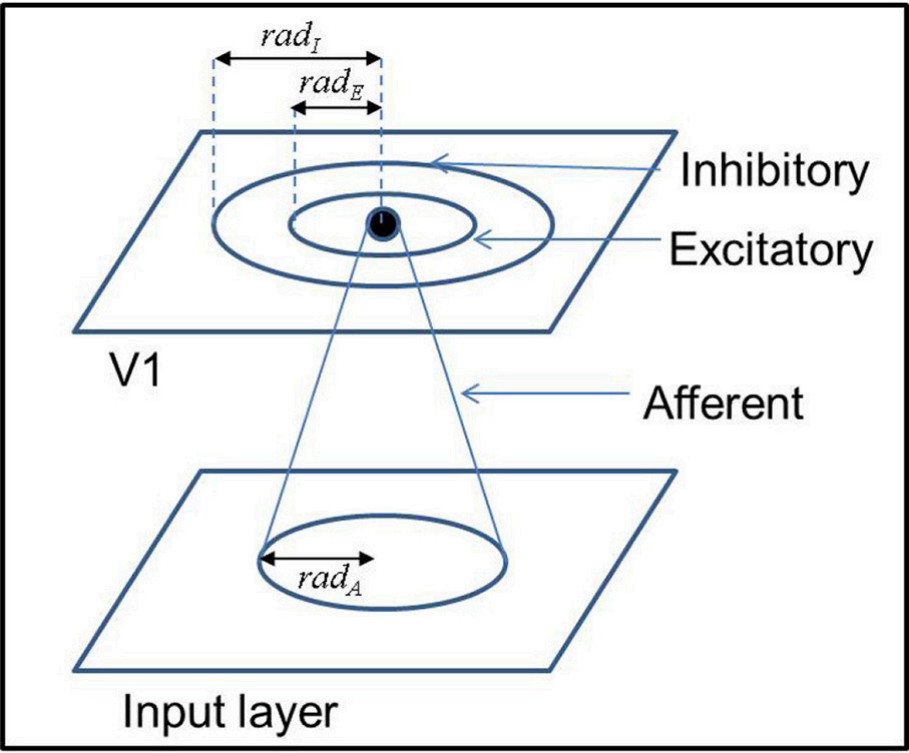
**A schematic representation of the architecture of the LISSOM model**.

A schematic representation of the LISSOM architecture is provided in Figure 1. The afferent and lateral weights are randomly initialized within the initial radius defined. The output of a particular neuron(*y*_*ij*_) in the output layer (V1), initially is dependent only on the afferent projections to that neuron as given by Equation (1).

(1) $$y_{ij}=g\left(\sum_{a,b}A_{ij,ab}x_{ab}\right)$$

where (*a, b*) denotes a neuron in the receptive field of the (*i, j*)^*th*^ neuron in the output layer, with input given as *x*_*ab*_; *A*_*ij, ab*_ represents the weight from the (*a, b*)^*th*^ neuron to the (*i, j*)^*th*^ neuron; *g* is a piecewise approximation of the sigmoid function given as:

$$g\left(s\right)=\{\begin{array}{ll} 0 & :s\leq \alpha_{l}\\ (s-\alpha_{l})/(\alpha_{u}-\alpha_{l}):\;\alpha_{l}< & \;s<\alpha_{u}\\ 1 & :s\geq \alpha_{u} \end{array}$$

where α_*l*_ and α_*u*_ are set to 0.1, and 0.65, respectively. After this initialization the lateral connections start contributing to the output (*y*_*ij*_(*t*)) which depends on the output from the previous iteration (*y*_*ij*_(*t* − 1)). Thus, the output (*y*_*ij*_(*t*)) is given as:

(2) $$\begin{array}{l} y_{ij}(t)=g(p\sum_{a,b}A_{ij,ab}x_{ab}(t-1)+q\sum_{k,l}E_{ij,kl}y_{kl}(t-1)\\ \;-\;r\sum_{k,l}I_{ij,kl}y_{kl}(t-1)) \end{array}$$

where *p*, *q*, *r* are scaling factors; *E*_*ij, kl*_ is the lateral excitatory weight from neuron (*k, l*) to neuron (*i, j*) and similarly *I*_*ij, kl*_ is the lateral inhibitory weight from neuron (*k, l*) to neuron (*i, j*) (values specified in Table 1). Thus, the afferent input to the node *y*_*ij*_(*t*) is given as $p\sum_{a,b}A_{ij,ab}x_{ab}(t-1)$, similarly the lateral excitatory input is given as $q\sum_{k,l}E_{ij,kl}y_{kl}(t-1)$, and the lateral inhibitory input as $r\sum_{k,l}I_{ij,kl}y_{kl}(t-1)$. The weight update rule for this unsupervised model, is a normalized Hebbian, and is the same for afferent as well as lateral weights, as given in Equation (3).

(3) $$w_{ij,mn}(t+1)=\frac{w_{ij,mn}(t)+\eta y_{ij}(t)P_{mn}(t)}{\sum_{mn}\left(w_{ij,mn}(t)+\eta y_{ij}(t)P_{mn}(t)\right)}$$

where *P*_*mn*_ is a generalized notation representing the pre-synaptic activity originating from the neuron (*m, n*); η is the learning rate. Thus, *P*_*mn*_ could represent a neuron in retinal layer with afferent weights, or a neuron in the V1 layer with either lateral excitatory or lateral inhibitory weights. These learning rates can be different for each of the connections: η_*A*_, η_*E*_ and η_*I*_ are the learning rates for the afferent, excitatory and inhibitory connections, respectively.

**Table 1:** **Parameter values chosen in the LISSOM model for the simulation of the meridional angle map**.

**Parameter**	**Value**
*p*	1.05
*q*	2.3
*r*	2.45
η_*A*_	0.5
η_*E*_	0.3
η_*I*_	0.11
*rad*_*A*_	1
*rad*_*E*_	0.03
*rad*_*I*_	0.55

The receptive fields of the afferent connections of each node in V1 are initialized to be centered around that particular node with a radius of *rad*_*A*_. Each node of the output layer V1 also has lateral excitatory and inhibitory connections with other neurons in V1 within a radii of *rad*_*E*_ and *rad*_*I*_, respectively. The weights of each of the connections are initialized to be uniformly random within the radii mentioned.

The training stimuli are presented in a pseudo-random fashion to the network. For every iteration (stimulus presentation) the network output activity is allowed to settle (9 sub-iterations) before the next input stimulus is presented. The activity of the network is re-initialized for each new input stimulus presented. The simulation is allowed to proceed for 600 iterations, by which duration the maps formed are quasi-stationary.

**Figure 2:**
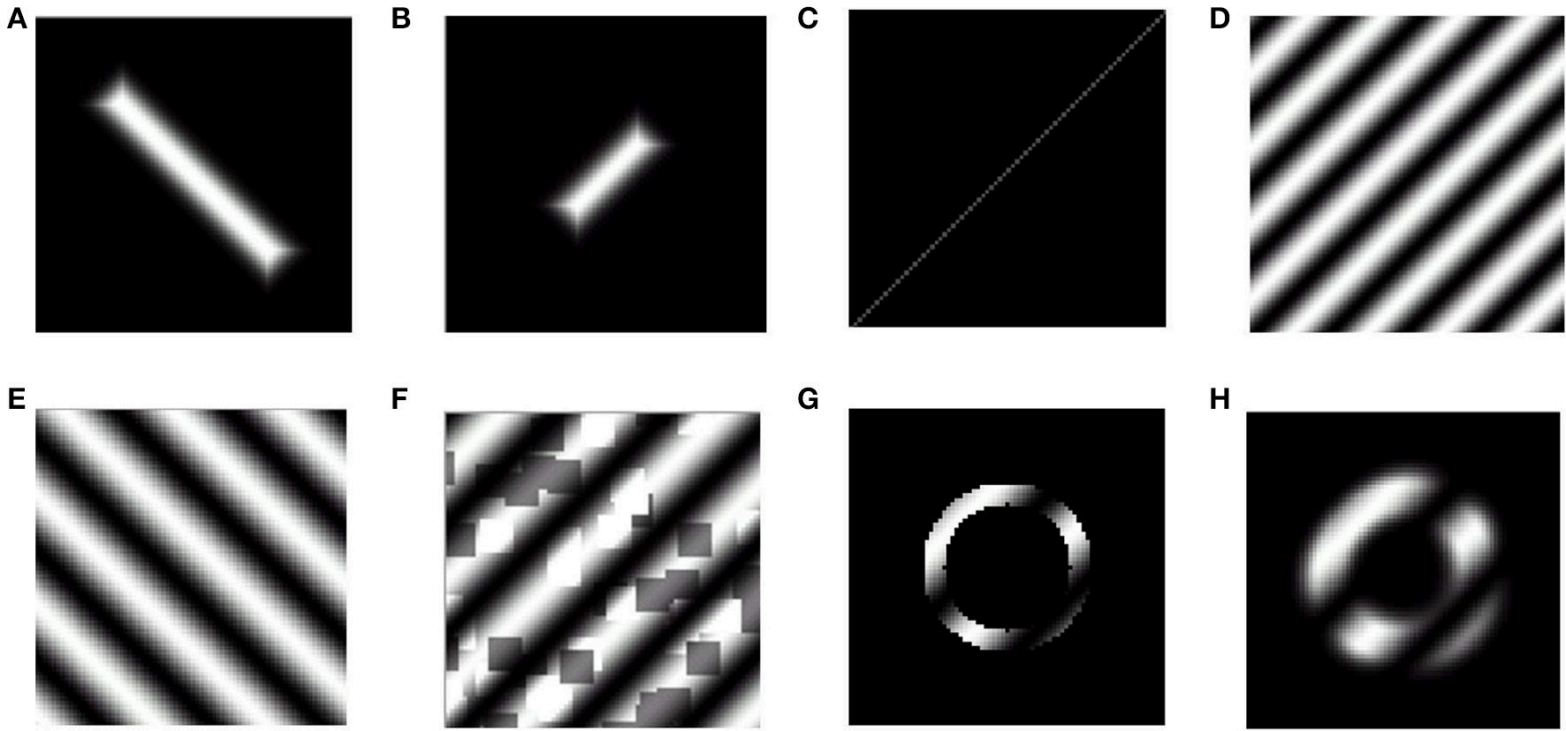
**Training and probing stimuli used in the LISSOM model**. The first 2 stimuli represent training stimuli, while the rest are representative of probing stimuli. **(A,B)** Rectangular bar with aspect ratio of 0.05, and randomized dilation and rotation; **(C)** Centered, collinear point stimuli to probe orientation preference; **(D,E)** Full field sinusoidal gratings with a spatial frequency of 0.75 cpd, 0.5 cpd to probe orientation preference; **(F)** Full field sinusoidal gratings with random noise added; **(G)** Annular ring subtending 2° of visual angle masked with a sinusoidal grating of randomized orientation and phase with a spatial frequency of 0.5 cpd; **(H)** Blurred edge annular ring subtending 2° of visual angle masked with a sinusoidal grating of randomized orientation and phase with a spatial frequency of 0.5 cpd.

This model is first trained on stimuli similar to those used in the development of the retinotopic map: which include rectangular bars of varying dilation and rotation (Figures 2A,B). It has been demonstrated that this training regime with an appropriate boundary condition yields a final map which closely resembles the retinotopic mapping of eccentricity and meridional angle onto orthogonal axes (Philips and Chakravarthy, 2015). This model is then probed to measure the orientation selectivity of each node in the output layer of the model which represents V1. The precise nature of the stimuli used for training as well as probing the map developed is described in the following subsection.

### Model stimuli

#### Training stimuli

##### Rectangular bars

One set of the stimuli employed for training are rectangular bars of varying dilations and rotations. The dilation (size) values vary from smallest 0.33° to largest 4° of visual field, as shown in Figures 2A,B. The rotation values vary from −π to π. The aspect ratio of stimuli used is 0.025.

#### Probing stimuli

##### Point stimuli

Collinear point stimuli with 24 different meridional angles are used to probe the development of the retinotopic map as shown in Figure 2C.

##### Full field sinusoidal gratings

In order to probe the orientation preference of a particular node in the output layer sinusoidal grating patterns which cover the entire visual field considered are used. The frequency of gratings used is 0.5 cpd or 0.75 cpd with only two orientations considered 45° and 135°. Each stimulus is presented for 18 different phases equally spaced between 0 and 2π. These stimuli are chosen to resemble the input stimuli used by Sasaki et al. (2006). Examples of such a stimuli are shown in Figures 2D,E.

##### Full field sinusoidal gratings with noise

In order to generate training and testing examples of the cortical responses corresponding to sinusoidal gratings with external noise, these gratings are masked with a random dot stereogram with a defined dot density (here 30%) as shown in Figure 2F.

##### Annular ring with sinusoidal gratings

Two different annuli are utilized, one subtending 2° of visual angle with a thickness of 0.285°, another subtending 2.285° of visual angle with a thickness of 2°. These annuli are masked with sinusoidal gratings having different spatial frequencies such as 0.5 cpd, 0.75 cpd. Each stimulus is presented for 18 different phases equally spaced between 0 and 2π. These stimuli are chosen to resemble the input stimuli used by Freeman et al. (2011). An example of such a stimulus is shown in Figure 2G.

##### Blurred edge annular ring with sinusoidal gratings

These inputs are similar to the annular ring stimuli, except that the edge of the ring is blurred with a smoothing parameter of 0.05. These stimuli are chosen to resemble the input stimuli used by Freeman et al. (2011). An example of such a stimulus is shown in Figure 2H.

### Model parameters and boundary conditions

The LISSOM model used simulates the central 4° of visual space and maps onto 27% of V1 (Adams and Horton, 2003). A constraint is imposed on the outer limit of the output layer, in order to approximate the shape of V1. It has been demonstrated that this boundary condition is vital in map development. If *u* and *v* represent the horizonal and vertical axes of V1, respectively, then the boundary of the cortical area is given as:

(4) $$u=log(\sqrt{a^{2}+{\left(a\tan(v)\right)}^{2}})$$

where *a* is a constant set to 1. A detailed derivation for this boundary equation is given in Philips and Chakravarthy (2015). Within this spatial boundary, the total number of V1 nodes considered in the simulation is 2945. The input space is discretized such that 24 nodes represent 4° of visual space. The model parameters are chosen so as to develop a retinotopic map which resembles the map in V1. The parameters chosen are within ranges which are biologically realistic. A detailed description of their biological correspondence is given in Philips and Chakravarthy (2015). All the simulations performed are using the Topographica Simulator (Bednar, 2009).

### Analysis

In order to analyze the results of the simulation, a number of measures are utilized. These measures have been previously used by Freeman et al. (2011) to analyze some of the experimental results which are compared in this study.

#### Map representation

**Figure 3:**
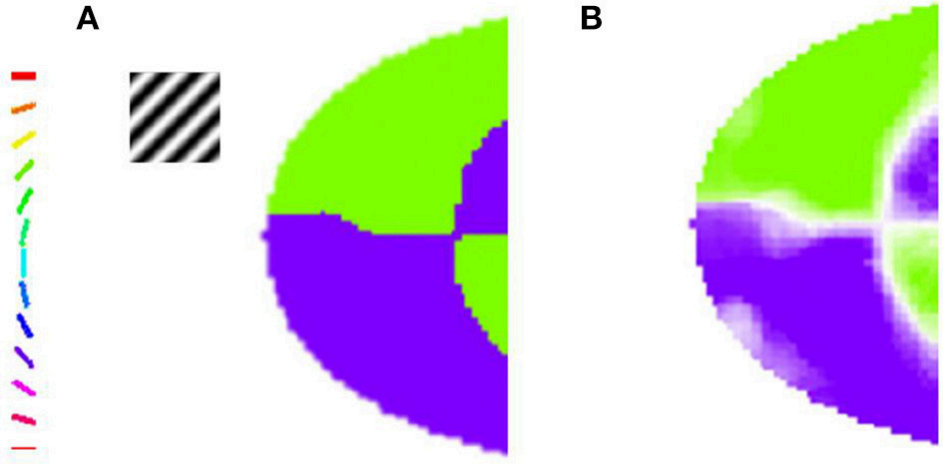
**The orientation map developed when the trained LISSOM model is probed with gratings having orientation 45° and 135°, respectively, with varying spatial frequencies**. The nodes which respond maximally to one of the orientations are color coded appropriately as shown in the colorbar. These results resemble (Sasaki et al., 2006), where the majority of nodes in the upper half of the output map prefer the 45° orientation, while the nodes in the lower half respond to the 135° orientation stimulus. **(A)** Orientation Preference of the map developed on probing with gratings of 0.5 cpd; **(B)** Combined Orientation Preference and Selectivity on probing with gratings of 0.5 cpd.

For a given stimulus, the feature (either orientation or meridional angle) which has the highest circular average across all the phases presented is assigned to a particular node in the output layer of the model. This feature is then color coded as shown in Figure 3. The number of colors present in the map equal the number of features probed.

#### Map similarity

In order to demonstrate the similarity between the meridional preference map and the orientation preference map two measures are utilized, namely the circular cross correlation, and the overall shift. The circular cross correlation for a node *n* with orientation preference *o* and meridional preference *m* is given as:

(5) $$r_{c}=\frac{R(o-m)-R(o+m)}{2\sqrt{\sum_{n\;=\;1}^{N}\sin^{2}(o_{n}-\bar{o})\sum_{n\;=\;1}^{N}\sin^{2}(m_{n}-\bar{m})}}$$

where *N* is the total number of nodes; $\bar{o}$ and $\bar{m}$ represent the circular mean of all orientation and meridional preferences, respectively; the concentration of angular sum (or difference) : *R*(*o*±*m*) is given as:

(6) $$R(o\pm m)=\left|\sum_{n\;=\;1}^{N}e^{i(o_{n}\pm m_{n})}\right|$$

where |.| is the magnitude of the complex number. The value of *r*_*c*_ ranges from −1 to 1, and greater the value better the circular correlation. When each corresponding *o*_*n*_ and *m*_*n*_ have similar values, indicating a close match between the 2 maps, *R*(*o*−*m*) becomes close to *N*. Similarly *R*(*o*+*m*) takes a value close to 0. Thus, the relative values of the 2 terms in the numerator dictates the measure of correlation. The denominator simply normalizes the value.

In order to test the statistical significance of the correlation a randomization test was performed. Thus, meridional angles and orientations of any one node were randomly assigned to any other node. This process is repeated 10,000 times to obtain the null hypothesis distribution such that the 2 maps are uncorrelated. The *p* value was calculated as the fraction of samples in the null distribution which were less than the actual *r*_*c*_ value.

Estimating the shift between the meridional and orientation maps involves computing Δ_*shift*_ where:

(7) $$m=o+\Delta_{shift}\text{modulo 2}\pi$$

The best value for Δ_*shift*_ can be achieved by minimizing the following function:

(8) $$\sum_{n=1}^{N}(\cos(m_{n})-\cos(o_{n}-\Delta_{shift}){)}^{2}+{\left(\sin(m_{n})-\sin(o_{n}-\Delta_{shift})\right)}^{2}$$

This optimal value ${\hat{\Delta }}_{shift}$ can be obtained by differentiating the preceding equation and equating to zero:

(9) $${\hat{\Delta }}_{shift}=tan^{-1}\left(\frac{\sum_{n\;=\;1}^{N}\sin(o_{n}-m_{n})}{\sum_{n\;=\;1}^{N}\cos(o_{n}-m_{n})}\right)$$

#### Classification

We wanted to verify if the orientation of the grating shown to the model could be deciphered from the LISSOM output (V1) response. The purpose of this exercise is to replicate results which demonstrate that fMRI data could be used to decode the orientation of the gratings shown to human and monkey subjects. For the classification task we construct a vector from all the individual nodes of the LISSOM output layer. The neuronal responses corresponding to sinusoidal gratings with a given cpd and randomized phase, masked with noise stimuli with a given dot density are considered as data vectors for the classification. There are 60 data vectors for each of the orientation bins (class) used for training, whereas the remaining 40 data points per class are used for testing. The data points are randomized and a Monte-Carlo cross validation is performed in order validate the accuracy over randomized train and test sets. The overall accuracy is calculated as the average accuracy over all cases. The model used for the classification is a multi-class support vector machine (SVM) (Hsu and Lin, 2002). A linear kernel is used for the SVM in order to estimate the optimal separating hyperplane.

#### Sufficiency

In order to demonstrate that the coarse scale orientation map developed was sufficient for orientation decoding, we divided the meridional angles possible into bins of width *w* and segregated the nodes on this basis. We then averaged the orientation preference of these nodes which lie within a single bin. The bin widths *w* were then varied and the classification accuracy was determined. We then compare this accuracy profile against that when nodes are assigned to a particular bin randomly.

#### Necessity

In order to evaluate the necessity of the coupling between the retinotopic map and the orientation map, we remove the component corresponding to the meridional angle of each node from its corresponding orientation preference. This is accomplished by calculating the residual ($\bar{R}$):

(10) $$\bar{R}=\bar{y}-\frac{\bar{y}\cdot \bar{x}}{\bar{x}\cdot \bar{x}}\bar{x}$$

where:

(11) $$\bar{y}={\left[\cos(o),\sin(o)\right]}^{T}$$

(12) $$\bar{x}={\left[\cos(m),\sin(m)\right]}^{T}$$

This ensures that the residual ($\bar{R}$) is orthogonal to the removed component *x*.

## Results

The global orientation map is developed on training the LISSOM model with dilated and rotated near radial rectangular bars as described in the Model Stimuli section. In order to verify the map developed a number of probing stimuli are used. The subsections detail the comparison of the map developed when compared with a number of experimental observations. The first subsection compares the map developed on probing with stimuli similar to the ones used by Sasaki et al. (2006). The second subsection verifies the map developed, on probing with stimuli similar to the one used by Freeman et al. (2011). The third subsection attempts to answer the question regarding the contribution of the global orientation map developed in orientation decoding. The last subsection attempts to elucidate the relationship between the global orientation map and the radial bias.

### Comparison with Sasaki et al.

In their paper, Sasaki et al. (2006) used 45° and 135° gratings. These grating are full field gratings. They observe that for the 45° grating the upper region responds maximally, whereas for the 135° grating the lower region does so. The map developed also shows the similar kind of responses as shown in Figure 3. These results are a subset of the results shown in the next section when the map developed is compared with experimental observations by Freeman et al. (2011). However, these results help verify that the global map is not a consequence of the boundaries of the stimuli used as the stimuli used are full field.

### Comparison with Freeman et al.

**Figure 4:**
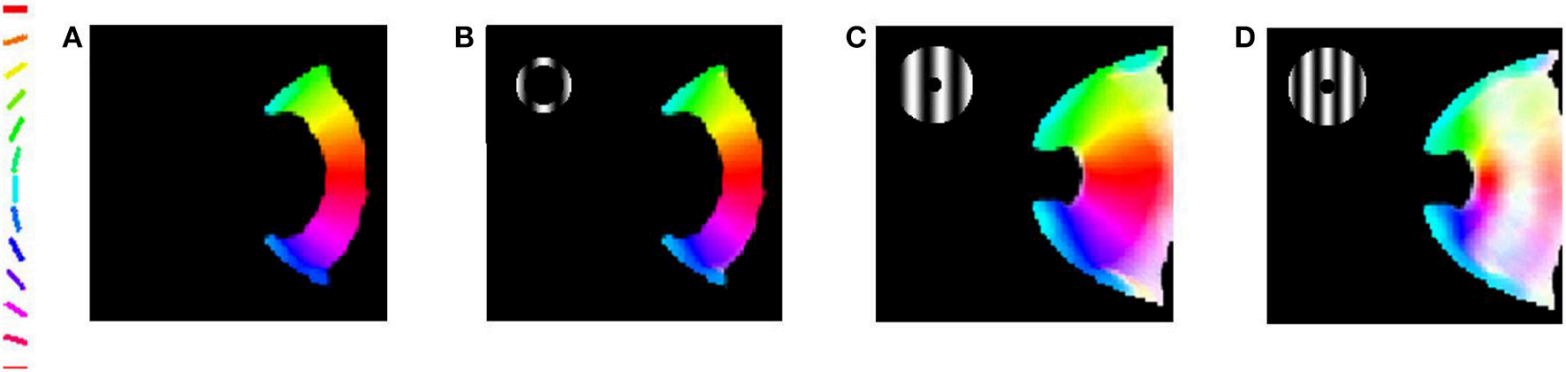
**The orientation map developed when the trained LISSOM model is probed with gratings having 12 orientations equally spaced between 0° and 180°, respectively**. The nodes which respond to these orientations are color coded appropriately as shown by the colorbar. These results resemble (Freeman et al., 2011) with the orientation map developed resembling the meridional map developed. **(A)** Meridional preference map developed; **(B)** Orientation preference map developed on probing with a thin annular ring with spatial frequency of grating set to 0.5 cpd; **(C)** Orientation preference map developed on probing with a thick annular ring with spatial frequency of gratings set to 0.5 cpd; **(D)** Orientation preference map developed on probing with a thick annular ring with spatial frequency of the grating set to 0.75 cpd.

**Figure 5:**
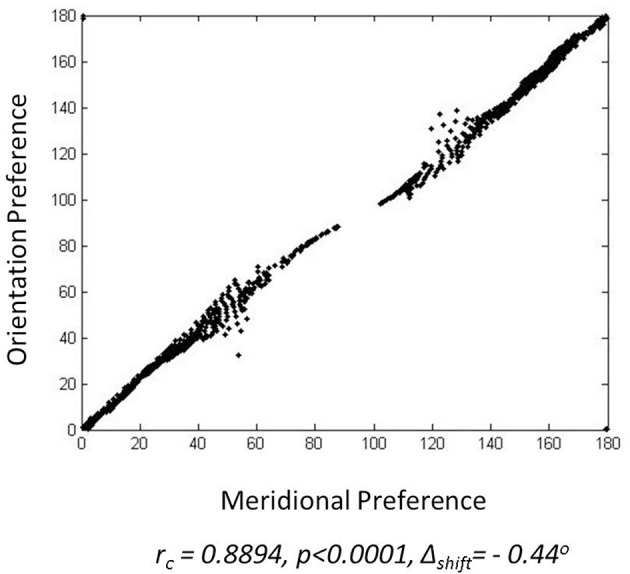
**Similarity between the meridional preference and orientation preference maps: For a single node the orientation preference is assigned to the y-coordinate where as the meridional preference is assigned the x-coordinate, so as to give the location of a point**.

The meridional preference and orientation preference maps developed in response to stimuli similar to those used by Freeman et al. (2011) are shown in Figure 4. One set of stimuli used are annular rings with sinusoidal gratings as described in the methods section. The nodes responsive to the 12 different orientations and 12 different meridional angles are appropriately color coded. The radial bias in the orientation map, almost mirroring the meridional map, is observed (Figures 4A,B). The orientation map developed (Figure 4B) on probing with a thin annular ring having a sinusoidal grating with spatial frequency of 0.5 cpd (as described in the methods section, shown in the inset) is compared with the corresponding meridional map developed (Figure 4B) on probing with an annular ring of the same thickness. The circular cross correlation (*r*_*c*_) between the meridional and orientation preference was found to be 0.8894 as shown in Figure 5. When compared with the corresponding *r*_*c*_ on randomization (described in the methods section),none of the 10,000 reshuffled data points exceed the *r*_*c*_ of 0.8894, giving a *p* < 0.0001. The Δ_*shift*_ between the 2 maps was determined to be −0.44°. These results are comparable with the experimental results described by Freeman et al. (2011) which have *r*_*c*_ = 0.52, *p* < 0.0001, Δ_*shift*_ = 1° for the corresponding maps developed using the same stimuli.

This effect appears to be robust, even on increasing the width of the annulus and increasing the spatial frequency of the gratings to an extent (Figures 4C,D). The selectivity, however is reduced as represented by the lighter shades of the map developed. The orientation map developed (Figure 4C) on probing with a thick (almost full field) annular ring having a sinusoidal grating with spatial frequency of 0.5 cpd (as described in the methods section, shown in the inset) is compared with the corresponding meridional map developed on probing with an annular ring of the same thickness. The similarity measures of *r*_*c*_ = 0.6357, *p* < 0.0001, Δ_*shift*_ = −0.9122° for the simulation are comparable with experimental results on using the same stimuli (*r*_*c*_ = 0.54, *p* < 0.0001, Δ_*shift*_ = 0°). However, it is to be noted that the experimental results correspond to only the peripheral region of interest (ROI), whereas the simulation results consider the complete maps developed.

Interestingly, on increasing the spatial frequency and considering a full field input the experimental *r*_*c*_ falls to 0.28 (*p* < 0.0001), with negligible change in the shift between maps (Δ_*shift*_ = 0°). The simulation results also show a similar trend on increasing the spatial frequency. The orientation map developed (Figure 4D) on probing with a thick (almost full field) annular ring having a sinusoidal grating with spatial frequency of 0.75 cpd (as described in the methods section, shown in the inset) is compared with the corresponding meridional map developed on probing with an annular ring of the same thickness. The *r*_*c*_ value now falls to 0.5007 (*p* < 0.0001), and the shift between maps developed is Δ_*shift*_ = −0.7965°.

**Figure 6:**
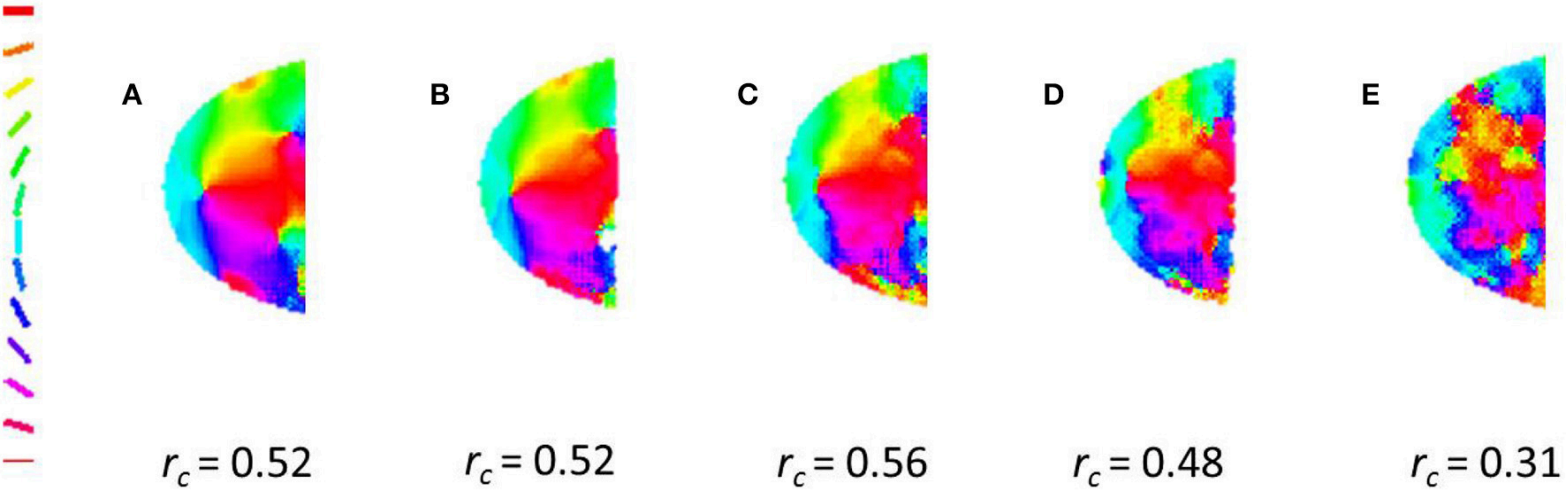
**A radial bias is observed even on training with different ratios of radial to non-radial orientation**. For the maps shown **(A)** 1 in 2, **(B)** 1 in 3, **(C)** 1 in 5, **(D)** 1 in 10, **(E)** 1 in 20 of the orientations given for their training are radial. The cross-correlation values are given the bottom of each map.

In order to ascertain the influence of non-radial stimuli in the development of global orientation map, the model is trained with varying ratios of radial to non-radial input stimuli. The circular cross-correlation values between the meridional and orientation maps are determined. A 0.5 cpd grating is used to probe the orientation map developed. We observe a decent cross-correlation even when only one in ten stimuli are guaranteed to be radial (See Figure 6).

**Figure 7:**
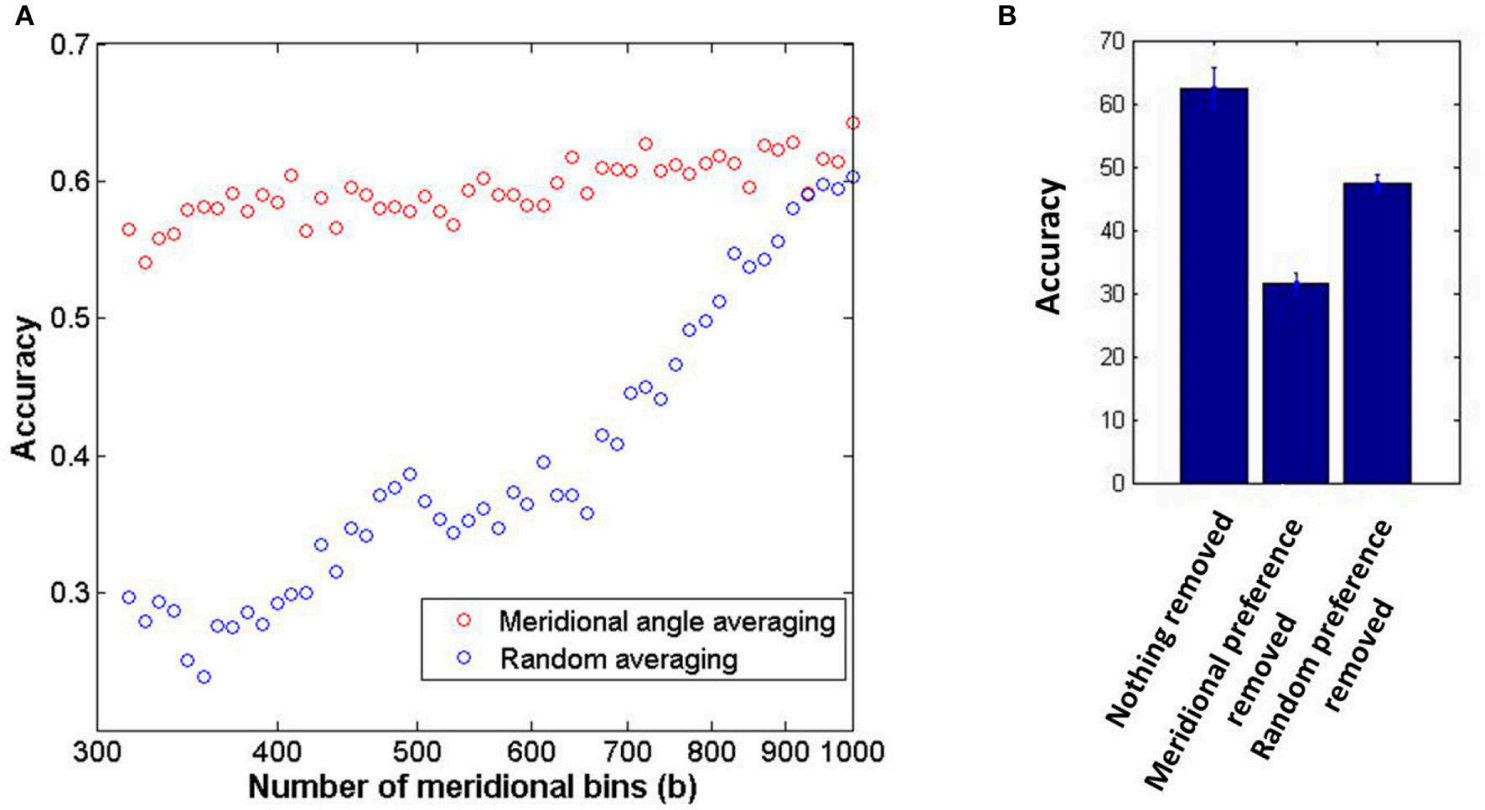
**The coarse scale orientation map is sufficient in order to achieve orientation discriminability from the output activity of the model**. **(A)** The sufficiency of the orientation map to classify orientations is demonstrated by circular average of the orientation of nodes which have similar meridional preference as opposed to random averaging; **(B)** The necessity of the radial bias is probed by removing the component corresponding to meridional preference from the orientation preference of each node as opposed to removing a random meridional component.

The neuronal responses corresponding to orientation gratings masked with external noise are probed in order to perform the classification task as described in the methods section. It should be noted that in this subsection the decoding results shown correspond to the thin annular ring containing sinusoidal gratings with spatial frequency of 0.5 cpd alone. The accuracy in determining the orientation preference from the LISSOM output activity is 62.46% (10% external noise added to stimuli) for 12 classes. The sufficiency and necessity of the radial bias in the decoding of input orientations are probed for in Figure 7. The dip in accuracy on removal of the meridional preference from the orientation preference of each node demonstrates the contribution of the radial bias in orientation discriminability; however it is still above chance and hence does not guarantee necessity. The experimental results report an accuracy of 54% without removing any component (Freeman et al., 2011). This number drops to 20% on removing the meridional component. However, on removing a random component from the orientation preference the accuracy increases to 43%. The tolerance to averaging out the orientation preferences of nodes having similar meridional preference as compared to random averaging, demonstrates sufficiency. Experimental results show a similar trend with accuracy dropping to 40% on meridional angle based averaging as opposed to 10% on random averaging (Freeman et al., 2011). There is a caveat to be kept in mind however. The demonstration of the sufficiency of the radial bias by averaging the orientation preference of nodes which have similar meridional preference, assumes that the orientation map developed must be locally similar to the meridional map. Similarly, removing the meridional component from the orientation preference assumes a point to point correspondence between the two maps. While this assumption certainly appears to be true for the maps developed corresponding to the stimuli considered in this subsection (thin annulus, 0.5 cpd), for maps corresponding to stimuli with higher spatial frequencies and wider annuli, this assumption is invalid (as apparent from the decreasing *r*_*c*_ values). This now begs the question: In the absence of such a clean node to node correspondence between the two map, is orientation decoding still possible using the orientation preferences developed?

### The radial bias and orientation decoding

As described in the previous subsection, as the spatial frequency and annulus width of the stimuli shown are increased, the *r*_*c*_ value between the orientation map and the meridional map developed decreases. However, this does not imply that the orientation map developed is devoid of features which may be useful in order to decode orientational information using fMRI data alone. The orientation decoding accuracy using the orientation maps developed for various stimuli are shown in Tables 2–4. As expected in each of the cases the performance does not worsen on decreasing the number of classes (orientations) considered. Interestingly, even though there is a dip in the *r*_*c*_ value of the maps developed for thick annulus 0.5 cpd stimuli as compared with to that for thin annulus 0.5 cpd stimuli, the accuracy is actually higher. This suggests that the node to node correlation between the orientation and meridional maps may not be necessary for orientation decoding. On increasing the spatial frequency (Thick annulus 0.75 cpd), the accuracy decreases as the number of classes considered is increased. However, most experimental results demonstrating high orientation discriminability for such high frequency stimuli, only consider a limited (≤ 6) number of classes. Tong et al. (2012) report accuracy levels of 80% for distinguishing responses to orthogonal stimuli for 0.25 as well as 1.0 cpd gratings. The accuracy drops to 70% for a pair of 4.0 cpd gratings. These results suggest that a more loose mapping between the orientation map and the meridional map, than the point to point mapping conventionally assumed, may exist. At higher spatial frequencies, the cross correlation between the 2 maps reduces. However, as far the model is concerned, the orientation maps developed, irrespective of the stimulus used, is a consequence of the training regime of radial bars employed in order to develop the meridional preference of each node.

**Table 2:** **Orientation decoding accuracy (10 times Monte-Carlo cross validated with standard deviation given in the bracket) on varying the probing stimuli, with 10% noise density**.

**Number of orientation classes**	**0.5 cpd thin annulus (%)**	**0.5 cpd thick annulus (%)**	**0.75 cpd thick annulus (%)**
2	100 (0)	100 (0)	98.25 (2.05)
6	95.87 (1.20)	97.93 (0.94)	68.96 (1.86)
8	86.12 (2.24)	88.69 (1.77)	50.72 (2.01)
12	62.46 (3.11)	68.00 (2.40)	33.37 (1.68)

**Table 3:** **Orientation decoding accuracy (10 times Monte-Carlo cross validated with standard deviation given in the bracket) on varying the probing stimuli, with 30% noise density**.

**Number of orientation classes**	**0.5 cpd thin annulus (%)**	**0.5 cpd thick annulus (%)**	**0.75 cpd thick annulus (%)**
2	100 (0)	100 (0)	97.25 (2.48)
6	92.00 (1.42)	92.54 (1.18)	60.33 (1.46)
8	78.31 (2.80)	82.87 (2.49)	44.59 (2.15)
12	53.94 (1.99)	57.54 (1.30)	26.79 (1.40)

**Table 4:** **Orientation decoding accuracy (10 times Monte-Carlo cross validated with standard deviation given in the bracket) on varying the probing stimuli, with 50% noise density**.

**Number of orientation classes**	**0.5 cpd thin annulus (%)**	**0.5 cpd thick annulus (%)**	**0.75 cpd thick annulus (%)**
2	100 (0)	100 (0)	93.75 (3.17)
6	86.79 (1.54)	89.66 (1.04)	55.87 (2.91)
8	71.62 (1.71)	75.25 (3.01)	42.50 (2.53)
12	44.78 (2.19)	50.77 (1.99)	25.25 (1.32)

### The radial bias and the global orientation maps

**Figure 8:**
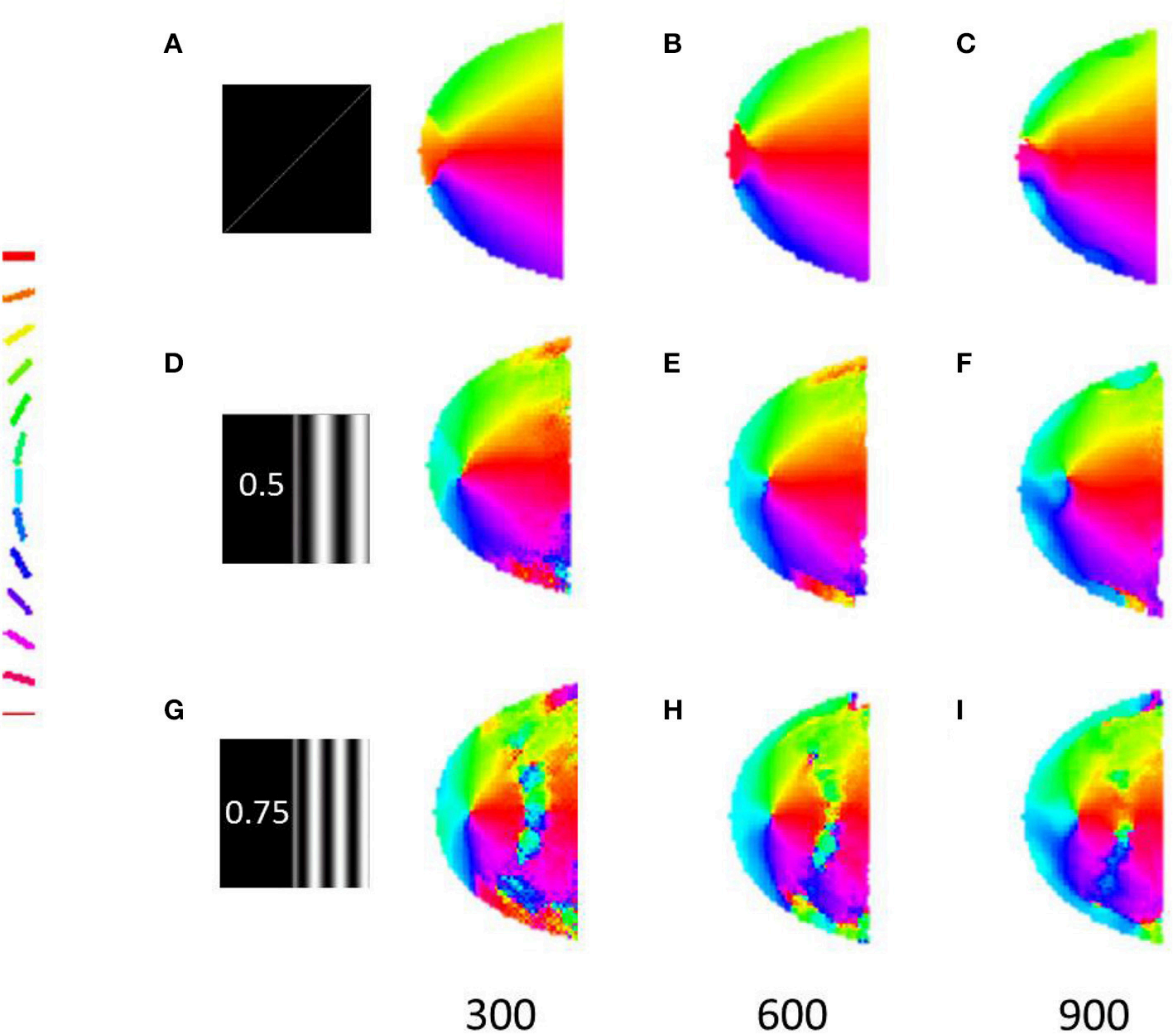
**Development of the meridional preference map and orientation preference maps**. Developing meridional preference maps when probed at **(A)** 200, **(B)** 400, **(C)** 600 iterations, respectively; Developing orientation preference maps when probed at **(D)** 200, **(E)** 400, **(F)** 600 iterations respectively with full field orientation gratings having a spatial frequency of 0.5 cpd; Developing orientation preference maps when probed at **(G)** 200, **(H)** 400, **(I)** 600 iterations respectively with full field orientation gratings having spatial frequency of 0.75 cpd.

**Figure 9:**
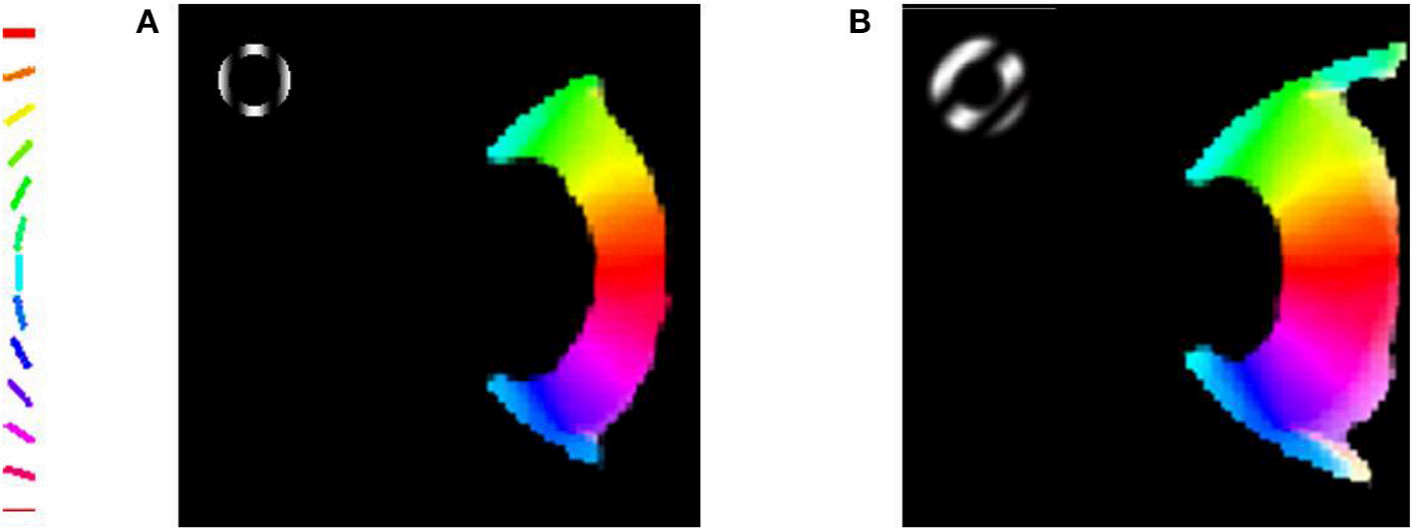
**The blurring of the edge of the annular ring does not seem to affect the radial bias**. **(A)** Orientation map developed on probing with a sharp edged annular ring masked with a sinusoidal grating; **(B)** Orientation map developed on probing with a blurred edged annular ring masked with a sinusoidal grating.

The robustness of the radial bias in the orientation map is checked for different spatial frequencies of full scale gratings, annular ring gratings with blurred edges. The map development across iterations for meridional preference as well as orientation preference for spatial frequencies of 0.5 and 0.75 cpd is shown in Figure 8. It is seen that as the spatial frequency is increased the corresponding orientation map begins to show prominent discontinuities. This result was also similar to that observed by Freeman et al. (2011). The effect of having a sharp edged or blurred edged annular ring as the probing stimulus does not seem to play a role in the orientation map developed as shown in Figure 9.

**Figure 10:**
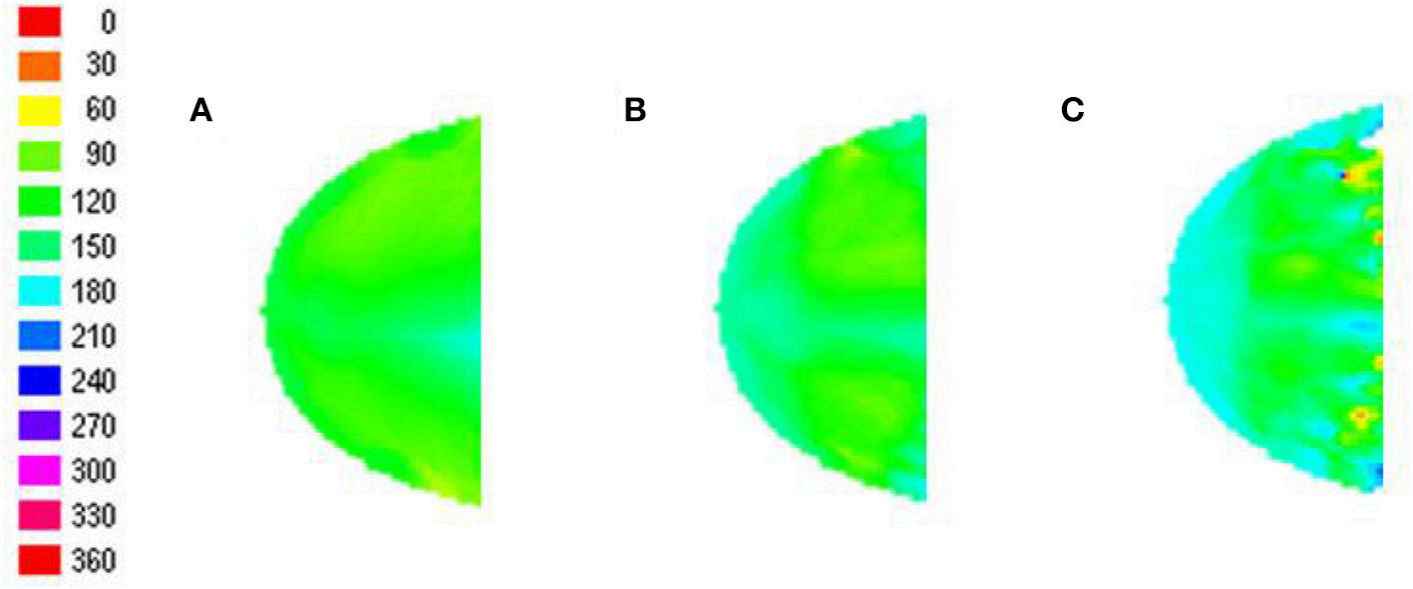
**The phase preference map developed reflects the preferences of individual nodes to that phase of the grating, when the middle peak of the grating matches with radial stimuli**. **(A–C)** represent the phase preferences on testing with sinusoidal gratings of varying orientation for spatial frequency of 0.25, 0.5, and 0.75 cpd, respectively.

A key insight from the model is that radial stimuli aid in the development of an orientation map which resembles the meridional map. The radial stimuli thus would also bias the phase preference of the global map developed. This leads to a testable prediction of the model: The presence of a global phase preference map across spatial frequencies as shown in Figure 10.

**Figure 11:**
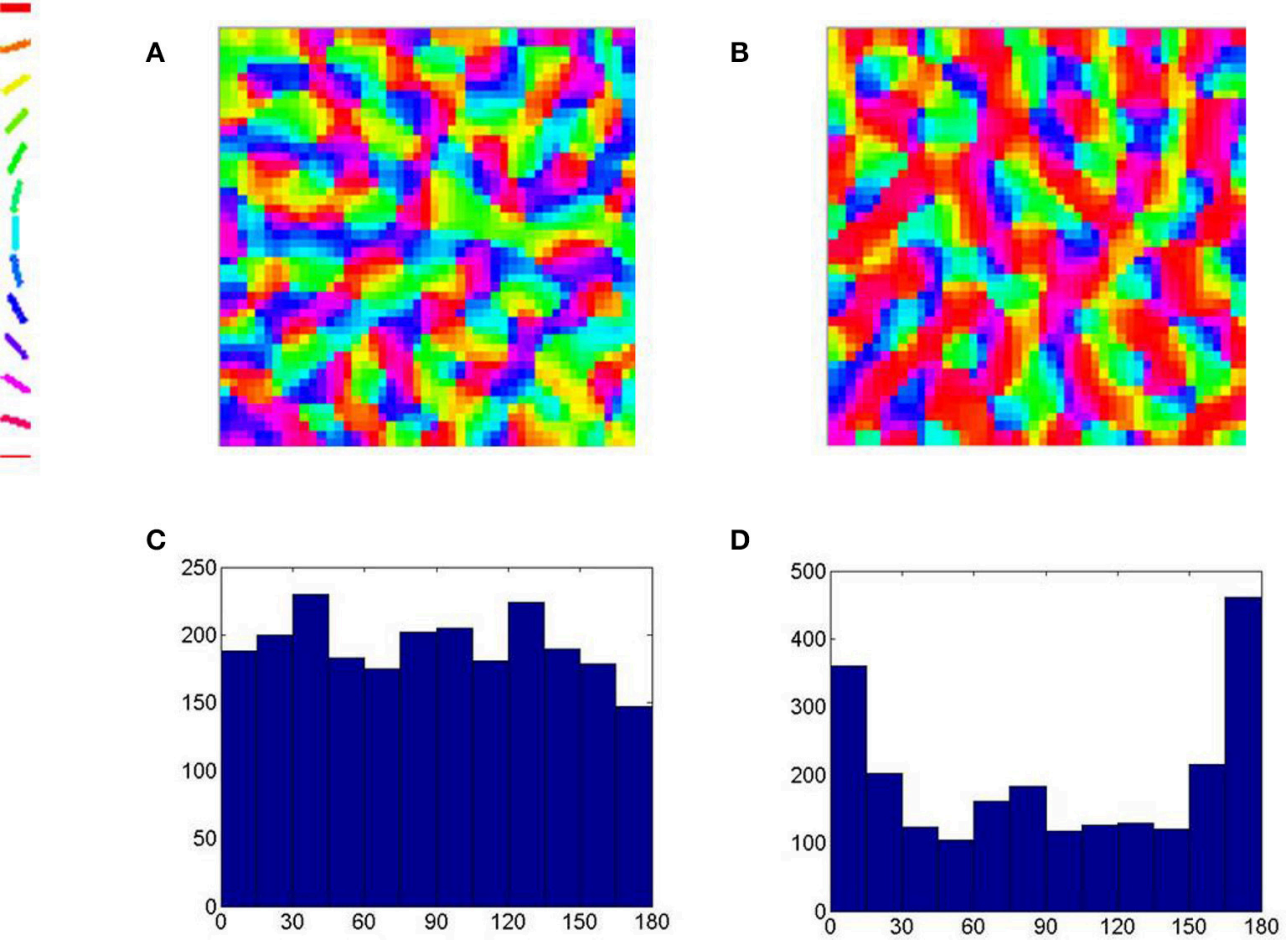
**The fine-scale orientation map developed at the scale of the hyper-column**. **(A)** The orientation map developed using equi-probable orientations for training and **(C)** the histogram of the orientations in the map. **(B)** The orientation map developed when one in 10 orientations used for training are horizontal (here radial) and **(D)** the histogram of the orientations in the map demonstrating an over-representation of the horizontal (here radial) orientations.

The scale of the simulation performed does not allow one to visualize orientation maps at the scale of the hypercolumn. It is however possible to simulate a sub-region of the map at the level of the hyper-column. An additional simulation is performed in which there is an over-representation of the horizontal (here radial) orientation in the input training regime. We observe the effects of such an input bias reflected in the map developed as well (See Figure 11). A region represented using a grid of size 48 × 48 neurons in order to observe the fine-scale orientation map developed. The radial orientation is assumed to be horizontal for the location under consideration. The same LISSOM model is trained with and without an input training bias of horizontal orientations. We observe a fine-scale orientation map with pin-wheels, fractures and iso-orientation domains in both cases. We also observe that in the map developed there is an over-representation of the horizontal (here radial) orientation when such an input training bias is employed.

## Discussion

A computational model which simulates the development of the radial bias is described in this paper. It is proposed that similar mechanisms that result in the development of the global retinotopy, could also create a global orientation bias, namely the radial bias using self organizing mechanisms. The Willshaw-von der Malsburg SOM model (Willshaw and Von Der Malsburg, 1976) was initially proposed to demonstrate that correlated activity in the input (retinal) layer, could result in a topography preserving map in the output (tectal) layer. A simplified (in terms of architecture) and more generalized (in terms of input dimensions) version of SOM was proposed by Kohonen (1990). Self organizing models and their variants have been used to simulate a number of features in the cortex including the responses of area V2 neurons (Plebe, 2012), the development of simple and complex receptive fields in V1 neurons (Antolík and Bednar, 2011), whisker direction maps in the barrel cortex (Wilson et al., 2010), the presence of tilt aftereffects in V1 neurons (Bednar and Miikkulainen, 2000), direction and orientation maps (Bednar and Miikkulainen, 2003).

In this study, the LISSOM model, when trained on dilated and rotated images has been shown to simulate the development of the global retinotopic map seen in primate V1 (Philips and Chakravarthy, 2015). In this paper it is further demonstrated that the same model on training with a stimulus set consisting of near radial bars also biases the development of a global orientation map. This implies that just as a there is complex-logarithmic transformation of retinotopy in the cortex, there also appears to be a complex-logarithmic transformation of a global orientation preference. We demonstrate that both these maps co-evolve given the appropriate stimuli and boundary conditions. In order to validate the results, the orientation map developed is compared with fMRI observations from Freeman et al. (2011) and Sasaki et al. (2006).

Sasaki et al. (2006), while first describing the radial bias, had speculated on the possibility of a link between the retinotopic and coarse scale orientation maps in V1. While modeling efforts have attempted to explain the difference between the angular preference and orientation preference around positive and negative singularities in the V1 of tree shrews (Paik and Ringach, 2012), no concrete model exists that simulates the global correlations between meridional angle and orientation preference. In this paper it is proposed that self organizing mechanisms on appropriate training could result in the development of the radial bias.

The LISSOM model maps the input features it receives such that nearby nodes in the output layer respond to similar features due to the short range lateral excitatory connections, whereas further away nodes respond to dissimilar features due to the longer range lateral inhibitory connections. Now when the model is trained using centered rectangular bars and provided the appropriate spatial constraint on the area of V1, the model maps the input space of eccentricity and meridional angle such that they are mapped on to orthogonal axes in the V1 layer (Philips and Chakravarthy, 2015). The rectangular bars used have an aspect ratio of 0.025, and hence are near radial. These stimuli now also cause the nodes of the V1 layer to have a preference toward radial orientations. This is because each node in the V1 layer, trained exclusively on near radial edges (because the bars are centered), using the Hebbian learning rule, evolves a receptive field which prefers such edges. Now when the orientation preference of each node of V1 is probed, a coarse scale orientation map emerges. The novelty of the results lies in the fact that just as meridonal angle gets mapped on to the horizontal axis in the V1 cortical space, orientation preference also gets mapped onto the same axis.

In order for a retinotopic map to emerge which resembles the retinotopy in V1, appropriate parameters are chosen to describe the connection strengths, learning rates and radii. These parameters are chosen such that they are biologically realistic. Similarly the boundary condition applied to the output layer such that it mimics the boundary of the primary visual cortex. A detailed description of role of the parameters and the boundary condition in the emergence of eccentricity and meridional angle being mapped on to orthogonal axes is given in a previous paper (Philips and Chakravarthy, 2015). Similar parameter and boundary conditions are used in this paper as well.

The preference of the global orientation map for orientations which are similar to the corresponding meridional angle is an indication of the radial bias. In order to quantify this similarity the two maps are compared to measure the circular cross correlation (*r*_*c*_) and the shift (Δ_*shift*_) between the orientation and meridional preferences of each node in the output layer of the model. These results are compared with experimental observation to validate a close match.

An additional insight that the modeling results provide is a plausible reason as to why the global orientation maps have a near one-to-one mapping for spatial frequencies between 0.25 and 0.5 cpd. Freeman et al. (2011), speculate this could be because of the mismatch between higher spatial frequencies and the receptive fields of individual neurons in the periphery. From the model it is apparent that, on moving toward the periphery of the visual field, the mismatch between the probing edges and the rectangular bar which was used for training would diverge as the spatial frequency increases. In other words, each node in the output layer of the model is probed for a larger number of edges at higher spatial frequencies many of which do not match with the receptive field learnt by that node during the initial training.

A key prediction of the model is the presence of a global phase preference map. This phase preference is a result of the radial nature of the stimuli used while training. Phase preference in V1 columns was thought to be randomly distributed, but Wang et al. (2015) in a recent paper describe the columnar organization of phase preference in V1. Just as Freeman et al. (2011) describe the presence of a global orientation map, we predict that there exists a global phase preference map which is prominent at 0.25–0.5 cpd.

It is to be emphasized that even though at higher spatial frequencies of input stimuli probed, the output orientation preference map does not correlate well with the meridional map, the orientation map developed still encodes a radial bias. It is likely that when such maps are used (corresponding to high frequency spatial patterns), a better than chance accuracy decoding of the orientation of the grating shown could be achieved, as demonstrated in the results section. This could explain why Kamitani and Tong (2005) report orientation decodability in the absence of an apparent global orientation map. The probing stimuli used by them had a spatial frequency of 1.5 cpd. Similarly Alink et al. (2013) have demonstrated robust decodability, on high pass filtering of V1 activity, suggesting that fine grained activity could also contribute to decodability. Again the spatial frequency of stimuli employed is 1.25 cpd.

In a recent paper, Pratte et al. (2016) state that the conventional radial bias is not necessary for orientation decoding on using grating stimuli of 0.5 cpd. They compute a measure called the retinotopy baseline, to quantify the retinotopic information present even after the removal of the conventional radial bias from the orientation preference. They report that on changing the stimuli parameters, duration protocol or removing harmonics, the difference between orientation decoding accuracy for radial bias removed orientation data and for the retinotopy baseline becomes significant. Their results also confirm that there is a drop in the accuracy on removing the meridional component, similar to the model results described earlier. As far as the model is concerned, removing the meridional component does not reduce the orientation decoding accuracy levels to chance.

Alink et al. (2013) also report robust discriminability on using globally similar but locally dissimilar stimuli such as patch swapped stimuli. Though the accuracies achieved are lower than for global stimuli, it is still significantly greater than chance. This led them to speculate that lateral and top down effects may play a role in orientation discriminability. Similarly context modulation effects may play a role in the global form effects which could also contribute to orientation discriminability (Cichy et al., 2015), suggesting the role of extra-striate feedback.

The current model does not capture neuronal spiking dynamics and is restrictive in its architecture. The scale at which the simulations are performed (the mapping of 4° of visual space), allows us to visualize only the large-scale bias in the orientation map developed even on training with non-radial stimuli. The current model also does not consider feedback from higher cortical regions. It would be a fruitful future endeavor to consider a more detailed model with top down feedback.

## Author contributions

RP: Computational model development, analysis, and manuscript preparation; VC: Computational model development, analysis, and manuscript preparation.

### Conflict of interest statement

The authors declare that the research was conducted in the absence of any commercial or financial relationships that could be construed as a potential conflict of interest.
